# Tendon, Ligament, and Muscle Injury, Osteotendinous, Myotendinous, and Muscle-to-Bone Junction Therapy Perspectives with Growth Factors and Stable Gastric Pentadecapeptide BPC 157—A Review

**DOI:** 10.3390/ph19020309

**Published:** 2026-02-12

**Authors:** Danijel Matek, Irena Matek, Mladen Japjec, Mirta Matek, Jakov Prenc, Borna Staresinic, Eva Staresinic, Andreja Prtoric, Suncana Sikiric, Lidija Beketic Oreskovic, Ivana Oreskovic, Sanja Strbe, Mario Kordic, Ante Tvrdeic, Sven Seiwerth, Predrag Sikiric, Alenka Boban Blagaic, Anita Skrtic, Ivan Bojanic, Ivan Dobric, Mario Staresinic

**Affiliations:** 1 Department of Pharmacology, School of Medicine, University of Zagreb, 10000 Zagreb, Croatiajakov.prenc@gmail.com (J.P.); abblagaic@mef.hr (A.B.B.); 2 Department of Surgery, School of Medicine, University of Zagreb, 10000 Zagreb, Croatia; 3 Department of Pathology, School of Medicine, University of Zagreb, 10000 Zagreb, Croatia

**Keywords:** tendon, ligament, muscle, osteotendinous junction, myotendinous junction, muscle-to-bone healing, therapy, growth factors, BPC 157

## Abstract

As a novel theoretical and practical advantage, preclinical to clinical evidence, this systematic review of PRP, growth factors, and stable gastric pentadecapeptide BPC 157 efficacy in complex musculoskeletal and junctional injuries emphasizes the cytoprotection concept, healing to restore tissue integrity. Notably, the concept holds tendon, ligament, and muscle healing, in particular. Then, it holds their healing together as interconnected lesions. Consequently, this review presents the possibilities for cytoprotective therapies suited for tendon/ligament/muscle and recovery of osteotendinous, myotendinous, and the muscle-to-bone junction. The estimated key was the success of injury recovery amid each agent’s direct exogenous administration, alone or with a carrier, locally or systemically, without reliance on complex scaffolds, carriers, or tissue-engineering constructs. As reviewed, while with commonly acknowledged physiological significance, and acting throughout cytoprotection principles, growth factors (PDGF, TGF-β1, IGF-1, FGF, VEGF, BMPs) delivered locally with various carriers improve tendon, ligament, and muscle healing; however, some (PDGF, TGF-β1, IGF-1) may fail in muscle lesions, and all show limited or no efficacy in junctional healing. Contrarily, proposed as a cytoprotection mediator, BPC 157 acts alone with a full cytoprotection range, given systemically or locally. Moreover, without any carrier, BPC 157 acts alone, combining beneficial effects on tendon, ligament, and muscle injuries with osteotendinous, myotendinous, and muscle-to-bone healing. In rat studies, across systemic (intraperitoneal, intragastric, or drinking water) and local (cream) administration, BPC 157 consistently demonstrated efficacy, indicating considerable translational potential. Further clinical studies will strengthen cytoprotective therapy and, particularly, BPC 157 in complex musculoskeletal and junctional injuries.

## 1. Introduction

No previous review has systematically evaluated tendon–ligament–muscle and junction healing therapies based on carrier-independent exogenous efficacy within a cytoprotection framework.

Tendon, ligament, and muscle injuries occur very frequently in sports medicine, along with damaged osteotendinous, myotendinous, and muscle-to-bone junctions. These are challenging problems in traumatology [1,2,3]. Especially in pharmacotherapy, these disturbances still require a solution [1,2,3]. It is possible that such pharmacotherapy could greatly benefit from the concept of cytoprotection [4,5,6,7,8,9,10,11,12] and its practical implementation (suited healing to restore tissue integrity and, therefore, the tendon/ligament/muscle and junction integrity [13,14,15,16,17,18,19,20,21,22,23]). Conceptually [4,5,6,7,8,9,10,11,12], cytoprotection agents (i.e., stable gastric pentadecapeptide BPC 157 [13,14,15,16,17,18,19,20,21,22,23]) occur with a large range of pleiotropic beneficial effects.

This review analyzed the degree of recovery of injury that could be achieved. This review pointed out the injury recovery by exogenous administration as a direct key and the possibilities of the tendon/ligament/muscle healing and junction healing as the particular effects and as the interconnected therapy effects. The focus was on growth factors [24,25,26], platelet-rich plasma (PRP) [27,28,29,30], and BPC 157 [13,14,15,16,17,18,19,20,21,22,23]. These effects on the injury recovery by exogenous administration of these agents, with carrier or alone, locally or systemically, as a direct key, were analyzed as special points of consequent relevance of growth factors [24,25,26] and stable gastric pentadecapeptide BPC 157 [13,14,15,16,17,18,19,20,21,22,23] in tendon, ligament, muscle, and junction healing. The stable gastric pentadecapeptide BPC 157 [13,14,15,16,17,18,19,20,21,22,23] appears as useful cytoprotective therapy. We performed a targeted literature search of PubMed/Scopus (years until October 2025) focusing on tendon/ligament/muscle healing, growth factors, platelet-rich plasma (PRP), and BPC 157. Conceptually, not only in theory [4,5,6,7,8,9,10,11,12], but in practice [13,14,15,16,17,18,19,20,21,22,23], true cytoprotection in the tendon, ligament, and muscle and junction should normalize the healing in either direction without causing opposite effects.

Therefore, as emphasized, the present review prioritizes demonstrable tissue recovery following direct exogenous administration as the principal indicator of translational relevance rather than exhaustive molecular pathway mapping, which remains incomplete for both growth factors and cytoprotective agents.

### 1.1. Growth Factor Background

Notably, the cytoprotection theory, providing its holistic application [4,5,6,7,8,9,10,11,12] and distinctive tissue healing, could be the resolving concept for the therapy of the tendon/ligament/muscle healing and junction healing. The original principle holds cytoprotection as the fundamental concept about preserving cell integrity under stress and against various noxious agents. Robert’s original gastric cytoprotection concept (1979–1983) and Szabó’s subsequent expansion emphasized several essential points [4,5,6,7,8,9,10,11,12]. There is the protection of cells from injury before structural damage fully develops. There is the preservation of membrane integrity and microcirculation. Modulation of inflammatory, oxidative, and vasoactive pathways is also postulated. The final point is restoring homeostasis across organs, even at distant sites (“general cytoprotection”) [4,5,6,7,8,9,10,11,12]. Also, this would be applied once the injury is already formed [4,5,6,7,8,9,10,11,12].

There are inherent cytoprotective actions [4,5,6,7,8,9,10,11,12], even if not described using that terminology. As a long-standing point, intended to overwhelm the reduction in inflammation and pain as a part of the effects of non-steroidal anti-inflammatory drugs (NSAIDs) and corticosteroids, there are various growth factors [31,32,33,34,35]. There are classic cytoprotective pathways described in Robert’s/Szabó’s original work [4,5,6,7,8,9,10,11,12]. Enhanced fibroblast proliferation, collagen organization, and neovascularization are the common findings in rodent or rabbit models of tendon, ligament, or muscle injury in studies carried out with platelet-derived growth factor (PDGF-BB), transforming growth factor-β1 (TGF-β1), insulin-like growth factor-1 (IGF-1), basic fibroblast growth factor (bFGF (FGF-2)), vascular endothelial growth factor (VEGF-A), and bone morphogenic proteins (BMPs) [31,32,33,34,35]. However, many studies [33,34,35] demonstrated histologic improvement, yet the healed tissue often failed to reach normal biomechanical strength. Likewise, mostly limited to direct local administration using specialized carriers or delivery systems, standard peptide growth factors elicit no full muscle recovery but rather scar tissue accumulation [36]. Thus, translation to clinical benefit has been limited. Human studies are mostly small, heterogeneous, or involve PRP rather than isolated recombinant growth factors. PRP serves as an indirect delivery system of multiple endogenous growth factors but shows variable outcomes in tendinopathies, muscle strain, and ligament repair [31,32,33,34,35]. Finally, growth factors have short half-lives and commonly require localized sustained release systems, delivery, and dosing as key limitations [31,32,33,34,35]. Also, overexpression (e.g., TGF-β1, bFGF, PDGF, IGF-1) can lead to fibrosis or disorganized collagen deposition, compromising function [32,33,37,38,39,40,41,42]. Thus, although growth factors demonstrate promising preclinical effects on fibroblast proliferation, collagen organization, and angiogenesis, these outcomes are regularly achieved using specialized carriers or delivery systems. Without such scaffolds, the rapid degradation and short half-life of growth factors significantly limit their therapeutic potential [31,32,33,34,35].

In contrast, BPC 157 exerts regenerative effects on tendon, ligament, and muscle in preclinical models regardless of the administration route, given alone, without requiring controlled-release matrices, highlighting a potential practical advantage over classical growth factor therapies [19,43,44]. This goes to the “therapeutic feasibility” argument, strengthening the comparison with BPC 157, by emphasizing that its efficacy is independent of carriers: the ease of administration, stability, and multi-tissue efficacy of BPC 157 may represent a practical translational advantage over conventional growth factor therapy [31,32,33,34,35], even if both remain mostly preclinical [13,14,15,16,17,18,19,20,21,22,23,43,44]. Thus, definitive clinical validation remains pending.

Therefore, this review aims to comparatively assess the regenerative and cytoprotective potential of the stable gastric pentadecapeptide BPC 157 in tendon, ligament, and muscle healing, with an emphasis on its mechanistic overlap and divergence from classical growth factors, and its translational prospects for sports medicine and trauma therapy. Notably, for growth factors, the failure to achieve full biomechanical recovery does not mean the lack of biological relevance.

### 1.2. BPC 157 Background

Along with all the challenges of conventional therapy non-steroidal anti-inflammatory drugs (NSAIDs), corticosteroids, and growth factors, carriers, and delivery systems, a particular challenge may be the stable gastric pentadecapeptide BPC 157, although most evidence is still preclinical [13,14,15,16,17,18,19,20,21,22,23,43,44]. In toxicology studies, BPC 157 exhibited a harmless limit test, 2 g/kg i.v. or i.g., without adverse effects in mice, and a lethal dose (LD1) was not achieved [13,14,15,16,17,18,19,20,21,22,23,43,44]. Therefore, these relations, important in practical evaluation, were particularly reviewed [13,14,15,16,17,18,19,20,21,22,23,43,44]. In addition, there is an increase in the number of users, although most evidence is preclinical. On the other hand, for BPC 157 therapy [13,14,15,16,17,18,19,20,21,22,23,43,44], human data are limited; it was effectively used in ulcerative colitis trials (phase II) without adverse effects and later in small studies, also without adverse effects, in knee pain and interstitial cystitis therapy [45,46,47,48]. Also, this includes favorable safety reports [49,50,51,52] comparable with previous notations (for more details, see [13]).

Notably, conceptual cytoprotective analogy generally comprises pleiotropic beneficial effects for high efficacy over standard agent therapy [4,5,6,7,8,9,10,11,12]. In particular, the high effectiveness of growth factors and BPC 157 and the distinction between BPC 157 and growth factors could be visualized in a cysteamine duodenal ulcer model in rat. On a molar basis, growth factors, such as PDGF, VEGF, and FGF, are about 2–7 million times more potent than the antisecretory drug cimetidine [53]. BPC 157 therapy seems to be even more effective. The molar effectiveness of BPC 157 highly exceeds that noted with PDGF, VEGF, and FGF [13,14,15,16,17,18,19,20,21,22,23,43,44,54,55]. Thus, important for tendon/ligament/muscle and junction therapy relation and outcome, being more (BPC 157) or less (growth factors) active in the cytoprotection concept implementation [13,14,15,16,17,18,19,20,21,22,23,43,44,54,55] would explain the limitations of the growth factor approach, carriers, and local application in tendon, ligament, and muscle injury therapy and the ban of junction therapy [31,32,33,34,35,56,57,58,59,60,61,62,63,64,65].

### 1.3. BPC 157 and Growth Factors: Cytoprotection Background

Since the initial introduction of the stable gastric pentadecapeptide BPC 157 in the early 1990s, as a peptide native and stable in human gastric juice and, thereby, easily applied as a novel mediator of cytoprotection, these pleiotropic beneficial effects have been largely reviewed [13,14,15,16,17,18,19,20,21,22,23,43,44,54,55]. Other groups also reviewed various aspects of BPC 157 [56,57,58,59,60,61,62,63]. Finally, there is an ability to rapidly activate collateral pathways to rescue perilous circumstances, otherwise leading to severe vascular and multiorgan failure [13,14,15,16,17,18,19,20,21,22,23,43,44,54,55]. Therefore, recently, stable gastric pentadecapeptide BPC 157 was presented as a therapy and safety key, with a special beneficial pleiotropic effect controlling and modulating angiogenesis and the NO system [13]. Wound healing and tendon, ligament, and muscle healing [19,43,44] were particularly shown in implementing the cytoprotection concept (i.e., cell protection as an innate stomach (gastrointestinal tract) mucosal maintenance and recovery to be translated to other organ therapy via cytoprotective agent application, cytoprotection→organoprotection [4,5,6,7,8,9,10,11,12]). In particular, there are seminal reviews of the effects of the stable gastric pentadecapeptide BPC 157 on musculoskeletal disturbances and all muscles in particular [19,20], elaborating this particular advantage by simultaneously engaging multiple pathways—cell proliferation, collagen synthesis, angiogenesis, and NO-system modulation, and cytoprotection—potentially essential for healing.

On the other hand, as a part of increased interest, recently, several reviews by other groups elaborated various aspects of the beneficial actions of the stable gastric pentadecapeptide BPC 157, particularly its effects on musculoskeletal health [64,65,66,67,68,69,70,71,72,73,74,75]. As they claimed, BPC 157 demonstrates promising preclinical efficacy, but robust clinical trials are lacking. The peptide may represent a novel therapeutic avenue [64,65,66,67,68,69,70,71,72,73,74,75]. On the other hand, without considering cytoprotection as a background for its pleiotropic beneficial effects but considering its large range of effects, there are current claims that it is a “panacea”.

While these reviews [64,65,66,67,68,69,70,71,72,73,74,75] also pointed out that BPC 157 shows consistent preclinical efficacy in tendon, ligament, muscle, and vascular repair, promoting angiogenesis and collagen synthesis [13,14,15,16,17,18,19,20,21,22,23,43,44,54,55], they attempt to resolve these points by indicating the tendon, ligament, and muscle as the particular lesions. However, in cytoprotection concept terms, all of them occur as interconnected lesions. Therefore, this review investigates the relevance of the stable gastric pentadecapeptide BPC 157 and other agents throughout not only their particular effects on tendon (Section 2), ligament (Section 3), and muscle injuries (Section 4) but also osteotendinous (Section 5), myotendinous (Section 6), and muscle-to-bone (Section 7) junction, amid their exogenous administration. More importantly, their relevance goes throughout their capabilities to provide integrative therapy of the tendon–ligament–muscle. These effects, achievements, and possible limitations were reviewed through the aspects of the corresponding conventional pharmacotherapies, particularly with respect to growth factor implementation and effects [31,32,33,34,35]. Notably, as a conceptual point that warrants further exploration, growth factors were also combined with the cytoprotection concept (i.e., PDGF-BB [76], TGF-β1 [77], FGF-2/bFGF [78], VEGF [79], IGF-1 [80], and BMPs [81]). Illustratively, IGF-1 is associated with cytoprotective effects in multiple tissues. It activates the PI3K/Akt and MAPK pathways, reducing apoptosis under stress conditions (oxidative stress, cytokine-induced damage). This is especially relevant in muscle and tendon cells [80].

However, as mentioned, growth factors lack the effect of direct exogenous administration as a key [19,43,44], amid the recovery of the injury itself. These effects are short-lived, not obtained with exogenous administration of the agents given alone, and depend on local delivery systems (i.e., carrier addition). Thereby, targeting one or several signaling pathways involved in tendon repair, such as PDGF-BB-mediated fibroblast proliferation [61,62], TGF-β1-driven matrix remodeling [41,63], and IGF-1-stimulated collagen synthesis [64,65], whatever is largely postulated as highly plausible for healing [31,32,33,34,35,56,57,58,59,60,61,62,63,64,65], may remain of limited translational relevance when not associated with demonstrable injury recovery. In contrast, the evidence that BPC 157 appears to integrate multiple reparative mechanisms simultaneously—angiogenic, tenogenic, myogenic, and cytoprotective—may be clearly combined with its beneficial effect, injury recovery, since obtained by native peptide application, whatever route of administration, without requiring a carrier [13,14,15,16,17,18,19,20,21,22,23,43,44,54,55]. Accordingly, this review prioritizes demonstrable tissue recovery following exogenous administration as the most relevant indicator of translational therapeutic efficacy.

Therefore, with these considerations, the mentioned complex molecular pathways behind the growth factors [31,32,33,34,35,56,57,58,59,60,61,62,63,64,65], as well as those shown to be involved in the effects of BPC 157 therapy [82,83,84,85,86,87,88,89,90,91,92], were not specifically reviewed. This also includes those molecular pathways involved in BPC 157 therapy’s counteraction of leaky gut syndrome [91] as well as counteraction of tumor-induced muscle cachexia [92], where it functions as both a stabilizer of cellular junctions and a free radical scavenger.

Thus, the leading approach is the consideration of the agent’s realized healing effect (i.e., recovery of either of tendon, ligament, or muscle lesions or recovery of all of them). It serves as proof of its efficacy by its exogenous administration. This provides that the agents, if indeed effective in tendon healing, should share the same effect on muscle healing or ligament healing as well. Notably, the value of exogenous administration for a particular agent’s innate efficacy could be quite distinctive. This could be given the administration of the agent alone (i.e., BPC 157) or in combination (i.e., growth factors), locally (i.e., growth factors) and/or systemically (i.e., corticosteroids, NSAIDs), need for special delivery systems or various carriers [93]. The proof of the valuable concept behind shows an effective cytoprotective principle; a shared regenerative mechanism, tendon healing, ligament healing, and muscle healing would proof each other healing. If such healing was successful, it would converge into successful junction healing.

Notably, in the mentioned BPC 157 reviews authorized by other groups that recently appeared [64,65,66,67,68,69,70,71,72,73,74,75] as well as in those reviews reviewing growth factors’ effects [31,32,33,34,35,56,57,58,59,60,61,62,63,64,65], the cytoprotection concept and the cytoprotective perspective were not considered. As a consequence, this healing-based review would be important, given that no previous review has systematically evaluated tendon–ligament–muscle and junction healing therapies based on carrier-independent exogenous efficacy within a cytoprotection framework.

Thus, this review provides a comparative, cytoprotection-based evaluation of musculoskeletal and junction healing therapies grounded in carrier-independent exogenous efficacy rather than isolated molecular signaling

## 2. Tendon Therapy

### 2.1. Background

Notably, the tendon disease pathophysiology and the challenges of achieving functional regeneration were extensively reviewed (while BPC 157 therapy was commonly not included) [31,32,33,34,35,56,57,58,59,60,61,62,63,64,65]. A common conclusion is that there is currently no pharmacological approach to augment the tendon healing process [93]. Pharmacologic therapies seek to modulate pain/inflammation, possibly support matrix repair, or use biologic agents based on the general estimation of tendon healing, inflammation, proliferation, and remodeling [60,94,95]. Likewise, there is a pathology of chronic tendinopathy, involving matrix degeneration, altered collagen structure, microruptures, neovascularization, and a dysregulated inflammatory response rather than classic acute inflammation [96,97,98].

### 2.2. NSAIDs, Corticosteroids, Nitroglycerin

Therefore, pharmacologic therapies offer short-term relief but may impair tendon healing with prolonged use. There are NSAIDs—systemic/local [99,100] and corticosteroids (local injections, systemic use less common, longer-term outcomes are not superior, and there is risk of tendon weakening or rupture if injected into or very near the tendon) [101,102]. This calls for novel biological and regenerative treatments capable of promoting true tissue repair [102]. Topical nitroglycerin (glyceryl trinitrate patches/gels) is proposed to increase local nitric oxide (NO), promote collagen synthesis, and improve tendon healing [103].

### 2.3. PRP and Stem Cell Therapies

PRP represents a transitional bridge between traditional pharmacotherapy and newer biologics (like peptides) that aim to regulate tissue regeneration at the molecular level. PRP delivers a concentrated source of autologous growth factors (e.g., PDGF, VEGF, TGF-β, and IGF-1) and cytokines that stimulate angiogenesis, fibroblast proliferation, and collagen remodeling. Clinical results are variable—while some trials report improved pain and functional outcomes, systematic reviews note inconsistent efficacy, likely due to differences in preparation and dosing protocols [104,105,106].

Stem cell therapies include tendon-derived stem cells, mesenchymal stem cells, or bone marrow-derived progenitors to contribute to matrix regeneration, anti-inflammatory modulation, and angiogenic support. Preclinical studies show promising results, but human data remain limited and heterogeneous. Regulatory and standardization challenges persist [107,108,109].

### 2.4. The Issue of Carriers

On the other hand, there is the issue of carrier and delivery systems. PRP is typically autologous and liquid, but its bioavailability and retention at the target site can be improved with carriers. There are fibrin gels/scaffolds to form a gel or clot, enhancing local retention and sustained growth factor release; hydrogels (collagen, gelatin, alginate) can encapsulate PRP, allowing controlled release and improved mechanical stability at the injury site; or matrix-based carriers provide structural support for tendon or ligament repair [105]. Similarly, there are many carriers used with stem cell applications [108,109].

Numerous carriers (i.e., diverse PRP preparation + diverse carrier(s) = diverse complexes) might create particular problems. As a point that is underestimated, there is an uncertain attribution of the resolving activity.

With growth factors, there is also the issue of carriers. While BPC 157 as a therapy was always given alone and, thereby, having undisputed activity attribution [13,14,15,16,17,18,19,20,21,22,23,43,44,54,55], the use of carriers seems to be essential for growth factor application and their therapeutic effect [31,32,33,34,35,56,57,58,59,60,61,62,63,64,65]. This may make the activity attribution of growth factors uncertain (peptide or carrier, or peptide + carrier). Likewise, this introduces uncertainty when attempting to integrate the growth factor effects reported in the corresponding studies because each formulation (peptide combined with different carriers) represents a distinct peptide–carrier complex (peptide + various carriers = various complexes of peptide + carrier) (Table 1).

Thus, while the efficacy of traditional growth factors often reflects the behavior of the carrier–growth factor complex, BPC 157 exerts its effects without such dependence, ensuring direct and unambiguous therapeutic attribution.

### 2.5. Classical Growth Factors (PDGF-BB, TGF-β1, VEGF, FGF, and IGF-1)

Growth factors have long been considered key modulators of tendon healing, based on their well-characterized cellular and molecular effects, fibroblast proliferation, chemotaxis, angiogenesis, and collagen synthesis [31,32,33,34,35,56,57,58,59,60,61,62,63,64,65]. Contrarily, direct experimental evidence for full tendon repair after exogenous administration remains limited (Table 2). From this perspective, most studies on tendon healing focus on theoretical or mechanistic aspects, relying on surrogate markers such as cellular activity, matrix deposition, and vascularization, assessed through in vitro assays or indirect in vivo measures, rather than directly demonstrating functional recovery of tendon lesions following exogenous interventions [31,32,33,34,35,56,57,58,59,60,61,62,63,64,65].

With these considerations, with a novel fibrin/heparin-based delivery system for sustained release designed to promote fibroblast proliferation and collagen remodeling at the repair site, PDGF-BB improves canine flexor tendon repair [62]. This need for optimal delivery should also be seen with a crucial aspect of tendon healing and repair and the evidence that PDGF-BB can enhance tendon cell proliferation in vitro [111]. Direct functional proof of tendon healing after TGF-β administration is likewise limited. Via adenovirus-modified muscle grafts, localized TGF-β1 delivery significantly improved tendon healing in a rat model and holds promise for clinical application [41]. Likewise, there are TGF-β3-loaded nanoparticles for tendon healing in a rat model [113] and adenoviral-mediated overexpression of TGF-β3 (ad-TGF-β3) in a rat model of flexor tendon injury [114]. This should also be seen with the evidence that TGF-β plays a dual role in tendon healing by promoting repair processes such as collagen synthesis and cell proliferation while also contributing to fibrosis and adhesion formation if not properly regulated [115]. IGF-I was incorporated into a collagen sponge, which was then applied locally to the tendon injury site, meaning localized, sustained delivery of IGF-I directly to the healing tendon tissue, Achilles tendon repair in rats [116] (note, delivery strategies were summarized [78]). Further support is intratendinous application in a horse [117] and human [118]. Contrarily, a randomized, double-blind, placebo-controlled clinical trial in patients with chronic patellar tendinopathy [119] found that intratendinous IGF-1 injection (1 mg) combined with heavy slow resistance training did not confer superior structural or clinical outcomes compared to training with saline injection. Notably, bolus injection of bFGF exhibited increased type III collagen/cell proliferation but no clearly improved strength or advanced repair quality [120,121]. This was later demonstrated by an FGF-2-coated nanofiber scaffold [122], showing how delivery strategies have evolved. Via intratendinous injection, tendon healing occurs with VEGF-A primarily via inducing angiogenesis, increasing vascular supply to support fibroblast activity and collagen deposition, which are crucial for tissue remodeling [123,124].

The efficacy of the application of growth factors stands on the assumption that local, sustained release is crucial for efficacy, given the tendon’s hypovascular environment and limited systemic drug access [31,32,33,34,35,56,57,58,59,60,61,62,63,64,65]. Even so, the growth factors’ efficacy, defined in this way, may be problematic. Notably, one essential healing effect, angiogenesis, with growth factors [124] would be beneficial in hypovascular/avascular tendon healing; it can be detrimental in other tissues, i.e., avascular cornea, and corneal neovascularization [125,126,127,128,129,130,131,132,133]. Moreover, there is a threat that agents like VEGF or bFGF enhance angiogenesis and early healing but induce uncontrolled or prolonged vascular stimulation that may impair collagen maturation and long-term tendon strength [121,133,134,135,136,137]. Importantly, in addition to tendon healing [19,43,44], BPC 157 induces corneal ulcer healing, counteracts corneal neovascularization, resolves corneal transparency, and results in corneal healing “angiogenic privilege” [18,23,138]. Likewise, it counteracts pathologic angiogenesis in cirrhosis and portal hypertension [18,23,139]. Thus, important for tendon healing, BPC 157 induces a self-modulating angiogenic response—facilitating early vascularization as shown before—that may be necessary for fibroblast survival and collagen deposition, unlike maintaining pathological neovascularization observed with prolonged VEGF or bFGF exposure [18,19,23,43,44].

Together, there is a lack of direct evidence of exogenous administration for the claimed therapeutic benefits of the growth factors. This pitfall would provide a general limitation that favors an alternative possibility, local vs. systemic application, carrier and special system delivery use vs. native peptide therapy application. Therefore, this can be a certain attribution of the tendon recovery to exogenous administration of the given agent, not limited only to its local (intratendinous) application. Likely, pleiotropic agents like BPC 157 may offer this advantage by simultaneously engaging multiple pathways—cell proliferation, collagen synthesis, angiogenesis, and cytoprotection—potentially replicating the combined effects of classical growth factors in a single molecule [13,14,15,16,17,18,19,20,21,22,23,43,44,54,55]. Providing the consistent effect of tendon healing combined with BPC 157 administration, these might be of considerable practical value.

### 2.6. BMPs

The other possibility for tendon therapy could be BMP therapy and the ossicle formation that could be noted with BMP therapy (Table 3) [140,141,142,143]. The local delivery via injection, fibrin gel, or collagen-coated sutures is considered to be critical to concentrate the effect at the repair site and reduce systemic exposure, which means the same limitations for BMPs studied in tendon healing, incorporating isoform, tendon effect, and delivery method, where there is a risk of ossicle formation. BMP-2 and BMP-7 are primarily osteogenic, with a higher risk of heterotopic ossification or ossicle formation within tendons [140,141,142,143]. BMP-12, -13, and -14 are considered more “tenogenic,” promoting tendon repair while minimizing bone formation [140,141,142,143]. The risk of ossicle formation is dose-dependent and influenced by the BMP isoform and carrier.

Thus, while BMPs (especially BMP-2, BMP-7) have repeatedly caused ossicle formation or ectopic bone within or adjacent to tendon tissue in animal studies [140,141,142,143], bFGF, VEGF, PDGF, IGF-1, and TGF-β do not directly induce ossicle in tendon models, though excessive angiogenic (bFGF, VEGF) or fibro-chondrogenic (TGF-β) signaling could indirectly favor calcification under pathological conditions. Illustratively, bFGF mainly promotes angiogenesis and fibroblast proliferation; however, excess or prolonged signaling can delay collagen maturation and indirectly support osteogenic microenvironments under certain conditions [110,134]. Angiogenesis can be coupled to osteogenesis/heterotopic ossification (a mechanistic link that makes vascular stimulation a potential indirect risk factor for ossicle formation) [144,145]. VEGF/angiogenesis can shift mesenchymal cell behavior toward aberrant differentiation (osteochondral pathways) in some contexts [146]. Thus, in contrast to classical growth factors requiring local carriers and facing short-lived effects or ossicle formation, BPC 157 demonstrates reproducible, systemic, and local tendon healing without ossification, highlighting its pleiotropic regenerative potential. In this context, considering BPC 157/BMPs distinction, in BPC 157 therapy, tendon–tendon continuities were reported to have re-established well, with no ossicles forming in other tissues [83,84,147,148,149,150,151,152,153]. As pointed out, with BMPs [154,155,156], the initial tendon healing process is misleading due to its similarity to the process of fracture healing [156] and the formation of ossicles in other tissues [154,155,156]. Thus, also in this respect, pleiotropic agents like BPC 157 may offer, in tendon healing, an additional way of healing [147,148,149,150,151,152,153], simultaneously engaging multiple pathways—cell proliferation, collagen synthesis, angiogenesis, that ossicle induction did not appear, and cytoprotection—potentially replicating the combined effects of classical growth factors and BMPs in a single molecule [13,14,15,16,17,18,19,20,21,22,23,43,44,54,55].

### 2.7. BPC 157

Thus, for the particular purpose of alternative tendon therapy (Table 4), the BPC 157 tendon evidence summarized in the preclinical literature (rodent in vivo tendon transection/detachment models + ex vivo/in vitro tendon cell work) [147,148,149,150,151,152,153] consistently reports that BPC 157 improves functional indices, biomechanical strength, collagen organization, and early revascularization and opposes corticosteroid-induced impairment of tendon healing [147,148,149,150,151,152,153]. Most in vivo tendon studies used systemic intraperitoneal dosing (daily; 10 µg/kg, 10 ng/kg, or 10 pg/kg tested) given in saline without a scaffold/carrier. In vitro/ex vivo studies applied BPC 157 directly to explants/cells. Therefore, BPC 157 therapy, as a native peptide therapy, successfully combines systemic and local therapy advantages [147,148,149,150,151,152,153] but evidently has higher efficacy than conventional growth factor and BMPs, which thereby need to be applied locally, either with a carrier or directly intratendinously [31,32,33,34,35,56,57,58,59,60,61,62,63,64,65,143,154,155,156]. As limitations, all tendon-specific functional/biomechanical evidence is noted in animal models (rats) and ex vivo/in vitro experiments. This should be confirmed in robust clinical trials in humans that are now lacking.

Therefore, it seems that the BPC 157 effect is consistent. BPC 157 accelerates functional recovery (e.g., Achilles functional index (AFI), muscle strength, range of motion), enhances biomechanical properties (load to failure, stiffness, Young’s modulus), and improves histological outcomes, including collagen organization and fibroblast alignment [147,148,149,153]. Mechanistically, BPC 157 acts on multiple levels: it stimulates tenocyte proliferation, survival, and migration; counteracts free radical formation; activates signaling pathways such as FAK–paxillin; enhances growth hormone receptor expression in tendon fibroblasts [83,84,149]; and promotes angiogenesis, which supports early tendon and ligament repair. Notably, it promotes angiogenesis in a particular way, given its beneficial effect on other tissues (i.e., cornea, liver) and inhibitory effect on corneal neovascularization, and pathologic angiogenesis during cirrhosis and portal hypertension [13,14,15,16,17,18,19,20,21,22,23,43,44,54,55], as mentioned before. Importantly, BPC 157 mitigates the negative effects of corticosteroids on tendon-to-bone healing and counteracts inflammation without impairing angiogenesis [147,148,149]. Overall, the data suggest that BPC 157 is a multi-modal, tendon- and ligament-targeted peptide that improves structural integrity, cellular viability, and functional recovery following tendon or ligament injury [83,84,147,148,149,150,151,152,153].

In summary, for tendon healing, tendon healing with the stable gastric pentadecapeptide BPC 157 occurs as a part of the broad pleiotropic activity that unites angiogenic modulation, cytoprotection, anti-inflammatory balance, and collagen organization within a single small peptide [13,14,15,16,17,18,19,20,21,22,23,43,44,54,55]. It promotes tendon fibroblast survival, enhances biomechanical recovery, modulates NO and growth-factor pathways, and accelerates functional healing without excessive vascular proliferation or fibrosis. This is unlike classical factors. Notably, they demand local scaffolds (note: the collagen sponge alone can stimulate a certain amount of regeneration [157], suggesting a possible biological synergy between the sponge and the growth factors). This also occurs when they are applied together (bFGF, BMP-12, and TGF-β1 loaded in collagen sponge [158]; IGF-1 and TGF-β1 in gelatin sponge as a scaffold for retention [159]). BPC 157 is effective alone after systemic (in vivo) or local (ex vivo/in vitro tendon cell work) administration and shows reproducible efficacy across diverse tendon-injury models [13,14,15,16,17,18,19,20,21,22,23,43,44,54,55]. It appears to have a self-modulating angiogenic profile and multilevel organ protection, suggesting a novel therapeutic avenue for regenerative medicine—potentially complementing or replacing multifactor cocktails currently required to mimic physiologic tendon healing [13,14,15,16,17,18,19,20,21,22,23,43,44,54,55] (Table 5). Further concept applicability occurs with demonstrated tendon healing in osteotendinous junction [147,148], myotendinous junction [160], and muscle-to-bone [161] junction recovery.

## 3. Ligament Therapy

### 3.1. General

Ligament healing parallels tendon healing: fibroblast proliferation, collagen synthesis, matrix remodeling, and angiogenesis are central. Classical growth factors require local delivery; systemic administration is largely ineffective [31,32,33,34,35,56,57,58,59,60,61,62,63,64,65]. PRP, a mix of autologous growth factors, accelerating healing in partial tears or reconstruction [162,163], and mesenchymal stem cells and ligament-derived progenitors that can enhance ligament regeneration [164,165,166,167] are promising, but clinical translation remains limited and heterogeneous. BMPs are less studied in ligament repair compared to tendons due to the risk of heterotopic ossification [168,169]. In ligament injury, BPC 157 therapy has the same effectiveness as in tendon healing [151].

### 3.2. Growth Factors in Ligament Healing

Classical growth factors—PDGF, TGF-β, IGF-1, and bFGF—play similar roles in ligament repair as in tendon healing (fibroblast proliferation, collagen synthesis, angiogenesis) [31,32,33,34,35,56,57,58,59,60,61,62,63,64,65]. Delivery vehicles are essential to improve growth factor and cell retention at ligament repair sites [31,32,33,34,35,56,57,58,59,60,61,62,63,64,65] (Table 6).

The most evidence in vivo [170,171,172,173,175,176,178] is that PDGF-BB and bFGF are the most consistently effective growth factors in enhancing ligament fibroblast proliferation, ECM synthesis, and biomechanical healing. In vitro evidence favors combination therapy (PDGF + bFGF + EGF + TGF-β) and synergistic effects, particularly in fibroblast outgrowth and ECM deposition [174,177]. In vivo, delivery methods, fibrin or collagen matrices, are essential to ascertain localized, sustained release, essential for the healing effect noted in PDGF-BB studies [170,171,172,173,175,176,178,179]. The other evidence is related to tendon healing for TGF-β [113,114], IGF-1 [115,116], bFGF [120,121,122], VGEF-A [123,124], BMP-12 [112,143], and BMP-2/BMP-7 [180]. Notably, tendons and ligaments share similar cellular compositions and healing mechanisms, including fibroblast proliferation, collagen synthesis, and extracellular matrix remodeling. Therefore, the tendon healing studies were assumed to ascertain ligament repair. However, this is in contrast with the evidence that rabbits’ standardized gap injury made in the medial collateral ligament had no benefit of TGF-β1 in a carrier solution applied into the right knee medial collateral ligament. There were no differences in the biomechanical measures, fibril diameter distributions, and histologic evaluation of the injured MCLs treated with TGF-beta1 or carrier alone [175]. No growth factor has translated into routine clinical ligament repair.

### 3.3. BPC 157 in Ligament Healing

To these points (local application into the ligament gap and carrier essential contribution), like in tendon injury healing, there is overwhelming evidence for BPC 157 that should be reviewed [151]. In rat medial collateral ligament transection, the BPC 157 treatment was BPC 157 administered intraperitoneally (10 µg or 10 ng/kg), per-orally via drinking water (0.16 µg/mL), or topically as a thin cream layer (1 µg/g). There was accelerated healing with improved functional recovery and enhanced biomechanical properties, including increased ultimate load. Likewise, BPC 157 improved collagen type I/III balance and enhanced vascularization early in the healing process. Thus, commonly, BPC 157 µg-ng-rats exhibited consistent functional, biomechanical, macroscopic, and histological healing improvements [151]. Furthermore, BPC 157 improved the healing of acute ligament injuries, and further ligament therapy could be substantiated, providing the matching effect of different application methods is commonly regarded as a highlight of the robust effect that could likely be translated.

## 4. Muscle Therapy

### 4.1. General

Muscle injury, seen very frequently in sports medicine, should also have a particular pharmacotherapy. This may be given the complexity of muscle injuries occurring by a variety of mechanisms, such as direct forces, including muscle lacerations and contusions, and indirect forces related to strains [181,182]. From a conceptual cytoprotection viewpoint, resolving of such complexity is implemented in the possible therapy effect.

### 4.2. NSAIDs and Corticosteroids

NSAIDs reduce inflammation and pain in acute muscle injuries but may impair satellite cell activation and myofiber regeneration by inhibiting COX-2-derived prostaglandins essential for muscle repair [183,184,185]. COX-2 inhibition after muscle injury delays muscle regeneration and reduces new muscle fiber formation [186]. Short-term NSAID use may be acceptable for pain control, but long-term use is detrimental to regeneration and hypertrophy [187]. Glucocorticoids inhibit myoblast regeneration and differentiation and increase muscle catabolism [188]. Chronic corticosteroid administration leads to decreased muscle mass and delayed repair after injury [188].

### 4.3. Growth Factors in Muscle Healing

Classical growth factors (IGF-1, bFGF, HGF, VEGF, TGF-β1) usually require carriers (microbeads, gels, hydrogels, or viral vectors) to maintain local bioavailability and avoid systemic degradation [31,32,33,34,35,56,57,58,59,60,61,62,63,64,65,189,190,191,192,193,194,195,196,197,198,199].

Contrarily, these pitfalls are largely avoided with BPC 157, which is carrier-free and stable in aqueous solution [13,14,15,16,17,18,19,20,21,22,23,43,44,54,55,200] (Table 7).

Classical growth factors (IGF-1, bFGF, HGF, VEGF, TGF-β1) typically require carriers such as microbeads, gels, hydrogels, or viral vectors to maintain local bioavailability and prevent systemic degradation. This creates the same fundamental limitation: the true effect of the peptide itself cannot be conclusively demonstrated. Notably, it is never applied alone and instead depends on specialized delivery systems. Likewise, in the case of muscle injuries, the healing effect of growth factors is also combined with local application.

Thus, for muscle healing, IGF-1 effectiveness needs local application (intramuscular) using engineered mRNA enabling localized, transient expression of IGF-1, enhancing regenerative potential and fiber formation more efficiently than protein alone [189,190]. For FGF to be effective and for its intramuscular application, these are cell-based or encapsulated systems providing sustained local delivery of FGF-2, protecting the factor from degradation and enhancing survival and integration of transplanted cells [191,192,193,194]. Intramuscular injection of HGF to enhance regeneration through improved satellite cell activation and favorable macrophage polarization provides mixed results in some models [195,196]. VEGF effects occur through adeno-associated virus (AAV) vector, collagen matrix, or VEGF-expressing stem cells; intramuscular injection; local application; or stem cell transplantation. Therefore, there is multifactorially improved muscle fiber regeneration and vascularization, reduced fibrosis, and enhanced functional recovery [197,198,199].

Likewise, the same carrier/delivery system limitation goes for a combination of multiple factors. Notably, regulating distinct aspects of the regenerative process (e.g., vascularization and stem cell activation) was used in parallel to affect the regeneration of functional tissues. This was the sustained delivery, via injectable gel, of a combination of VEGF to promote angiogenesis and IGF-1 to directly promote muscle regeneration and the return of muscle function in ischemic rodent hindlimbs [201].

Notably, the cytoprotection concept mandates the tendon/ligament/muscle healing congruence as an interconnected healing point and evidence that the agent that heals tendon lesions should also heal muscle lesions as well, and vice versa. It was demonstrated that PRP, which contains endogenous HGF, can mediate anti-inflammatory effects in injured tendons. Therefore, there is a suggestion that HGF plays a role in modulating the inflammatory response during tendon healing, while direct evidence based on the exogenous administration is lacking [202].

On the other hand, some growth factors do not align with such a cytoprotection agenda. Namely, unlike tendon and ligament studies, the direct evidence for exogenous PDGF administration for skeletal muscle fiber defect/tear healing remains sparse. Revascularization of ischemic tissues by PDGF-CC via effects on endothelial cells and their progenitors shows that daily treatment with PDGF-CC (4.5 µg/day) in a mouse femoral artery ligation (hind-limb ischemia) model enhanced skeletal muscle regeneration (more regenerating myocytes with central nuclei) and reduced necrosis [203].

This may be mechanistic complexity, given that PDGF signaling can be beneficial (proliferation, vascularization), but a persistent PDGF/PDGFRα signaling may lead to fibrosis in muscle (via FAP activation) [204].

Furthermore, in muscle healing, the tendon/ligament/muscle healing congruence as an interconnected healing point of the mentioned growth factors does not fit the apparent worsening that occurs with TGF-β administration [205,206]. As an illustration, exogenous TGF-β1 co-injected into glycerol-injured mouse tibialis anterior muscle significantly reduced myotube density and increased endomysial fibrosis and α-smooth muscle actin immunostaining, thus promoting fibrosis [204]. TGF-β1 treatment of injured muscle tissue induced fibrogenic rather than myogenic differentiation of muscle progenitors, promoting scar formation over muscle fiber repair, and overproduction in response to injury and disease is a major cause of tissue fibrosis in animals and humans [207,208,209,210]. Likewise, overexpression of IGF-1 has been correlated with muscle hypertrophy in transgenic mouse lines [42]. As a final statement that may be mostly limited to direct local administration, standard peptide growth factors elicit no full muscle recovery but rather scar tissue accumulation [36].

### 4.4. BPC 157 in Muscle Healing

Thus, with the unlimited applicability, using systemic application, and native peptide application, without any carrier addition, BPC 157 therapy may be a practical rescue in muscle healing, and thereby, the effects and way of activities are strongly distinctive from those described with growth factor application. BPC 157 therapy includes full transection of the quadriceps muscle [152,200], crush injury and corticosteroid systemic administration [211,212], muscle denervation [213], hind limb ischemia [85], and spinal cord injury [214,215] (Table 8).

#### 4.4.1. Specific Effects of BPC 157 in Muscle Healing

Therefore, since BPC 157 was always given alone [13,14,15,16,17,18,19,20,21,22,23,43,44,54,55,152,200,211,212,213,214,215], unlike the limitations noted in the growth factor effects [31,32,33,34,35,56,57,58,59,60,61,62,63,64,65,189,190,191,192,193,194,195,196,197,198,199], some specific points likely appear for the beneficial effects of BPC 157. There are muscle regeneration and angiogenesis, as BPC 157 consistently promotes angiogenesis, collagen alignment, and functional muscle recovery after severe injury [152,200]. The angiogenic response involves upregulation and modulation of VEGF and FGF2 [85,86,152], endothelial proliferation, and dense microvessel formation within regenerating muscle. Counteracting corticosteroid-induced impairment, where healing is typically suppressed, BPC 157 completely reversed the inhibition of angiogenesis and myogenesis [212]. Neuromuscular protection that appears after denervation, or spinal cord injury BPC 157 preserved muscle mass, mitochondrial integrity, and reduced oxidative stress [213,214,215] suggests a neurotrophic or metabolic stabilizing effect beyond local regeneration. Endothelial protection and NO signaling may appear as a background, as molecular studies [85,86] confirmed that BPC 157 activates VEGFR2 and eNOS, enhancing endothelial survival and microvascular repair (i.e., mechanistic cascade: BPC 157 → VEGFR2 → Src/FAK → Akt → eNOS → increased NO bioavailability, vasodilation, and revascularization). In conclusion, the consistent outcomes in transected, ischemic, and denervated muscle suggest a pleiotropic cytoprotective mechanism distinct from classical growth factors. In addition, BPC 157 is also effective when continuously confronted with repeated traumas [216,217].

As an important part of the applicability of these effects [13,14,15,16,17,18,19,20,21,22,23,43,44,54,55], the particular therapy effect occurs in mouse cancer cachexia, known as a serious metabolic syndrome affecting more than 50% of patients with advanced cancers, characterized by skeletal muscle atrophy, significant weight loss, loss of adipose tissue, and increased mortality [92]. BPC 157 mitigates breakdown and promotes repair/regeneration of muscle fibers and weight/muscle mass preservation, prolongs survival, counteracts catabolic pathways and promotes anabolic pathways, particularly through TNF-α and IL-6 inhibition, proteasome ubiquination inhibition, and anti-inflammatory action [92]. This should be combined with its particular modulatory effect on angiogenesis previously described as tissue injury specific (counteraction of corneal neovascularization, counteraction of pathologic neovascularization in cirrhosis and portal hypertension vs. advanced angiogenesis in other tissue healing, i.e., avascular tendon) [13,18,23,138,139]. Likewise, this should be viewed with the evidence that BPC 157 counteracts the VEGF effect and inhibits cell growth and VEGF signaling via the MAPK kinase pathway in the human melanoma cell line [218].

#### 4.4.2. BPC 157 Analgetic Effect

Furthermore, BPC 157 has an analgesic effect of its own [219,220,221,222,223]. This can be important not only for muscle injury healing and function recovery [92,152,200,211,212,213,214,215,216,217] but also for tendon and ligament injury healing [147,148,149,150,151,152,153] and combined healing, such as osteotendinous junction [147,148], myotendinous junction [160], and muscle-to-bone junction [161] healing, and wound healing in general [13,19,20,43,44]. As a regular effect of therapy, there is fast function recovery consistently upon injury induction, and the BPC 157-treated rats regularly exhibited a lack of leg contracture [147,148,149,150,151,152,153,160,161,211,212,213,214,215,216,217]. Thus, as pointed out [19], there may be lesser lesion and lesser pain, and the noted analgesic effect might be part of the therapy’s rapid healing effect. On the other hand, the use of the injured leg accordingly would improve the healing as well. Illustratively, and likely as a part of its particular healing effect, BPC 157 produced analgesia in the MgSO_4_ and acetic acid test in mice, a model of prolonged pain associated with tissue injury [219], and counteracted succinylcholine-induced muscle pain (violent screaming upon light touch) in rats [223]. Indicatively for the possible translation (i.e., multiple types of knee pain), 11 of 12 patients had significant improvement in knee pain after one intra-articular injection of BPC 157 lasting more than one year thereafter [47]. However, this might antagonize morphine analgesia and haloperidol potentiation of the morphine analgesia [224]. In addition, as mentioned [13,19,20], it might antagonize the effect of local anesthetics [225,226].

#### 4.4.3. BPC 157 Against Distinctive Etiopathology Muscle Disabilities and Weakness

Further, a point skipped to be discussed in this review is muscle therapy against distinctive etiopathology muscle disabilities and weakness [16,19,20]. This should be using the described beneficial effect in cachexia, as a highlight of the resolved complexity of the BPC 157 muscle healing effect (i.e., cachexia encompasses anorexia, hypoanabolism (reduced building of muscle/tissue), and hypercatabolism (increased breakdown)) [92]. Notably, in addition to tumor-cachexia [92], BPC 157 therapy might recover many muscle disabilities (i.e., succinylcholine [223], vascular occlusion [227,228,229,230,231,232,233,234,235,236], spinal cord compression [214,215], stroke [237], traumatic brain injury [238], severe electrolyte disturbances [239,240,241,242], neurotoxins (Parkinson’s-like disturbances in mice or multiple sclerosis-like disturbances in rats) [243,244], neuroleptics [245,246], alcohol [247,248], serotonin syndrome [249], and NO-system blockade [245]).

Thus, beyond local tissue regeneration, BPC 157 may restore global muscular and vascular integrity in systemic pathologies ranging from neurogenic atrophy to ischemic and metabolic syndromes, emphasizing its role as a broad-spectrum cytoprotective agent [16,19,20]. In a peptide analogy with IGF-1, and relevance of recovery of disabled muscle, as pointed out [250], several clinical trials have assessed the efficacy of systemic delivery of recombinant IGF-1 in patients who could benefit from strength gains. With mixed results, these include the aging population, patients with growth hormone deficiency, and those who suffer from amyotrophic lateral sclerosis and myotonic dystrophy [251,252,253,254,255,256,257,258,259,260,261].

Such a peptide analogy may be possible provided that both BPC 157 [13,14,15,16,17,18,19,20,21,22,23,43,44,54,55] and IGF-1 [80], as mentioned, share a cytoprotection background. However, given the extent of the beneficial effect, BPC 157 has more of it, while IGF-1 has less. Notably, in addition to the recovery of muscle disturbances, peripheral and central, BPC 157 therapy regularly recovers the primary lesions as well [16,19,20]. A shared illustration may be the recovery of stroke. BPC 157 given after initiation of reperfusion results in the recovery of primary insult neuronal/hypoxic in the hippocampus and the recovery of ischemia/reperfusion-induced substantial hippocampal neuronal injury. Behavioral deficits were substantially reversed, essentially approaching baseline (pre-surgery) levels in many tests [237]. Multiple sclerosis model, induced by neurotoxin cuprizone in rats [244], occurs with severe lesions. There were extensive cortical and subcortical necrosis, edema, and neuronal loss in the lesion area, hippocampus (CA1–CA4), and striatum, signs of microvascular destruction, perivascular hemorrhage, and neuronal chromatolysis. Severe motor and behavioral abnormalities were forelimb–hindlimb incoordination and ataxic gait and paresis or paralysis of contralateral limbs. BPC 157-treated rats, administered continuously in drinking water, showed consistent recovery. There were marked reductions in necrosis and edema, better preservation of cortical architecture, and survival of pyramidal neurons in the hippocampal CA1 region, with rapid and near-complete resolution of motor and behavioral abnormalities [244]. In rats with persistent hyperlithiemia, in lithium intoxication, muscle function recovery was combined with counteraction of the severe vascular and multiorgan failure; recovery of the brain, heart, lung, liver, kidney, and gastrointestinal lesions; counteracted thrombosis, centrally and peripherally; and whole Virchow syndrome [240]. In addition, the BPC 157 therapy might be the recovery for the disabled heart functioning [16,19,20] and disabled smooth muscle functioning (vessels [84] and various sphincter function [262,263,264,265,266]) recovery [16,19,20].

In summary, unlike growth factors that require local administration and carrier stabilization, BPC 157 consistently restores muscle structure and function through systemic cytoprotective, angiogenic, and neuroregenerative mechanisms—offering a unique, clinically translatable therapeutic profile.

Collectively, these findings indicate that BPC 157, through its multifaceted regulation of angiogenesis, cytoprotection, and neuromuscular restoration, represents a novel therapeutic class distinct from conventional growth factor-based muscle repair strategies. Further evaluation includes various junction recovery, specifically, osteotendinous [147,148], myotendinous [160], and muscle-to-bone [161] junctions.

## 5. Osteotendinous Junction Therapy

### 5.1. General

Generally, successful tendon-to-bone healing, essential for the overall outcome of surgical procedures aimed at repairing injured tendons, replicating the functional and biological characteristics of the native tendon–bone interface, as indicated in many reviews, remains challenging due to its limited regenerative capacity [267,268,269,270,271]. Basic-science research in orthopedics has explored multiple strategies to enhance tendon-to-bone healing, including osteoinductive growth factors, platelet-rich plasma, gene therapy, periosteal graft coverage, osteoconductive materials, cell-based therapies, biodegradable scaffolds, and biomimetic patches [267,268,269,270,271]. However, this review will focus on osteoinductive growth factors [267,268,269,270,271] and, on the other side, stable gastric pentadecapeptide BPC 157 [147,148] (Table 9). Namely, in reviews [267,268,269,270,271], BPC 157 as a therapy to rescue the osteotendon junction [147,148] was not specifically pointed out.

For model relevance, Rodeo 1993 describes the canonical histologic sequence in a bone tunnel model (dog) that is still widely used as the base model for testing augmentation strategies; augmentation studies typically place the BMP/GF on the tendon surface/repair site (often on a sponge/hydrogel/carrier) to increase local retention [270]. As an interesting point, this was commented on by Krivic [150].

Thus, the BMP family (BMP-2/4/7/12) presents multiple preclinical studies in rotator cuff, anterior cruciate ligament (enthesis), and Achilles contexts showing that the osteoinductive/chondro-inductive BMPs can promote bone formation and fibrocartilaginous enthesis features when delivered locally. As an essential point, carrier and release kinetics matter (collagen sponge, gelatin hydrogel sheet, brushite cement, and hyaluronan paste were all tested)—sustained, localized release generally produced better enthesis/early mechanical outcomes.

Besides the tendon-to-bone studies with FGF-2, an implied gelatin hydrogel sheet as the carrier system controlled the release of FGF-2 at the tendon–bone repair site [276,277].

No systemic growth factor therapy exists for tendon-to-bone repair.

### 5.2. BPC 157

On the other hand, as previously pointed out in tendon healing, in the osteotendinous junction recovery, BPC 157 therapy provides an alternative option [148,149,153]. BPC 157 in several rat studies using tendon detachment/transection (Achilles) and in a rotator cuff tear model reports functional, histologic, and biomechanical improvement after systemic (intraperitoneal) administration [148,149,153]. Likewise, the peptide countered corticosteroid-aggravated impairment of tendon-to-bone healing [148,149,153]. As an important point, the peptide was applied alone with systemic dosing. Carriers not required in these reports suggest a particular advantage over standard osteoinductive growth factor application and clear attribution to the peptide itself.

Furthermore, these effects on osteotendinous junction recovery should be viewed in the further therapy on myotendinous [160] and muscle-to-bone junction [161] recovery.

## 6. Myotendinous Junction

### 6.1. General

Generally, therapy of the severed myotendinous junction should be of particular interest [278]. Likewise, outcomes of operative and nonoperative management are well reviewed [279]. Notably, myotendinous junctions, as anatomical regions specialized in the transmission of contractile strength from muscle to tendon, are a common site where acute injuries occur. The transition zone between skeletal muscle and tendon, the myotendinous junction, has a key role in being the structure where muscle fibers interact with tendon [278].

Therefore, from the cytoprotection point of view, myotendinous junction therapy challenges the efficacy of both tendon therapy and muscle therapy, the capability to act alone in either tendon or muscle therapy, or together in myotendinous junction recovery. In practical therapy terms (growth factors vs. BPC 157 explained in this review), given the assumed unity of tendon–ligament–muscle healing within the frame of growth factors (i.e., PDGF, IGF-1, FGF, VEGF, TGF-β) or BPC 157 beneficial effects, the cure of the severed myotendinous junction would be the result of both effective tendon therapy and muscle therapy. These therapies should complement each other and prove each other’s efficacy, to give together the recovery of the muscle/tendon common structure (i.e., myotendinous junction) and reestablish the tendon muscle continuity and full function recovery (for review, see [19,20]).

### 6.2. PRP

Few reports of successful recovery of the severed myotendinous junction appeared with repeated ultrasound-guided PRP injections [280,281].

### 6.3. Growth Factors

In the myotendinous domain, without direct exogenous therapy studies at the myotendinous junction recovery, evidence of classical growth factors (IGF-1, TGF-β, FGF, BMPs) for myotendinous junction recovery goes with the acknowledged essential role of TGF-β/BMP and FGF signaling pathways in tendon development [282]. However, on the other hand, the evidence for therapeutic myotendinous junction repair remains mechanistic/developmental/exercise-modulation. During exercise, there is upregulated expression of IGF-1 and TGF-β or upregulated FGF but decreased TGF-β [283,284]. Thus, limited without direct exogenous therapy studies at the myotendinous junction, a mechanistic rationale linked to myotendinous junction plasticity for targeted therapies, along with tissue engineering constructs [285] that present those cues.

### 6.4. BPC 157

On the other hand, the stable gastric pentadecapeptide BPC 157 provides direct exogenous therapy studies at the myotendinous junction [160]. Myotendinous junction defect, which cannot heal spontaneously in rats, was the dissection of the quadriceps tendon from the quadriceps muscle in rats as a particular target to evidence consistent macro/microscopic, biomechanical, functional assessments, *eNOS*, and *COX-2* mRNA levels and oxidative stress and NO levels in the myotendinous junctions [160].

The matching beneficial effects were obtained with two ways of systemic application, using a regular BPC 157 protocol. BPC 157 (10 µg/kg, 10 ng/kg) regimen was given either intraperitoneally (first application immediately after surgery, last 24 h before sacrifice) or per-orally (in drinking water (0.16 µg/mL, 0.16 ng/mL, 12 mL/rat/day) until the sacrifice at 7, 14, 28 and 42 postoperative days). These BPC 157 regimens document prominent therapy effects (macro/microscopic, biomechanical, functional, much like *eNOS* and *COX-2* mRNA levels and counteracted oxidative stress and NO levels in the myotendinous junctions), while controls have a poor presentation. Especially in rats with the disabled myotendinous junction along with full functional recovery, BPC 157 counteracts muscle atrophy that is regularly progressive and brings muscle presentation close to normal. Accordingly, unlike the perilous course in controls, those rats, when receiving BPC 157 therapy, exhibit a smaller defect, and finally, defects completely disappear. Microscopically, there is no more inflammatory infiltrate; well-oriented recovered tissue of the myotendinous junction appears in BPC 157-treated rats at 28 days and 42 days [160].

In conclusion, BPC 157 restores the myotendinous junction in accordance with the healing of the transected muscle [200], tendon [147,148,149], and ligament [151]. Thus, with respect to the achievement of the healing of the severed myotendinous junction, there is restoration of muscle–tendon continuity, functional and mechanical recovery, healing effect involving anti-inflammatory and angiogenesis promptly achieved, systemic administration, in two ways, no carrier required. Together, these clearly suggest that BPC 157 therapy is congruent with tendon–ligament–muscle healing in the cytoprotection concept terms. Likewise, with myotendinous junction healing, the BPC 157 congruent therapy effect overcomes that of the growth factors. Therefore, unlike the BPC 157 large framework (including myotendinous junction healing), growth factors share a more limited framework of the tendon–ligament–muscle healing, only a framework that could be achieved without the possibility of myotendinous junction healing.

## 7. Muscle-to-Bone Healing

### 7.1. General

Stable gastric pentadecapeptide BPC 157 as therapy after surgical detachment of the quadriceps muscle from its attachments for muscle-to-bone reattachment in rats [161] should be seen with particular considerations. Aside from salvage methods, including the reattachment of various muscles, i.e., quadriceps [286,287], masticatory muscles [288], lateral pterygoid muscle [289,290,291], mentalis muscle [292], and temporalis muscle [293], as well as other procedures [294,295], very few studies address direct muscle-bone detachment/reattachment. The only classic study was the Chierici and Miller (1984) study of muscle reattachment following surgical detachment in rhesus monkeys [296]. The study detaches the temporalis muscle from bone and investigates reattachment, but no exogenous growth factor administration was applied [296].

Notably, the complexity of the cytoprotection concept is particularly challenging when implementing muscle-to-bone reattachment. Muscle-to-bone injuries, including surgical or traumatic detachment, represent a unique challenge distinct from tendon-to-bone or myotendinous junction injuries [296]. Muscle-to-bone and tendon-to-bone healing represent biologically and mechanically distinct processes [297,298]. In tendon-to-bone healing, the tendon, a dense fibrous connective tissue, reattaches to bone, typically forming a fibrocartilaginous enthesis; the primary challenge is re-establishing the tendon’s structural integration and tensile strength [296,297,298]. In contrast, muscle-to-bone healing involves direct reattachment of muscle fibers or muscle–tendon junctions to bone, which requires not only restoring mechanical continuity but also re-establishing viable myofibers, neuromuscular connectivity, and proper vascularization [296,297,298].

### 7.2. Growth Factors

Commonly, the reviews provide an overview of the biochemical and biomechanical interactions between muscle and bone, including myokines and osteokines, mechanical loading, and their parallel roles in musculoskeletal integrity and regeneration [299,300,301], while they do not address muscle-to-bone reattachment after detachment. Therefore, a solid mechanistic basis for understanding muscle–bone signaling relevant to the discussion of biological repair and reattachment processes remains with the absence of verified studies matching exactly muscle detachment from bone → exogenous growth factor therapy → reattachment to bone as a gap in the literature. Notably, there is preclinical work on muscle recovery (see Section 4) and tendon-to-bone healing (see Section 5), but the muscle-to-bone detachment model + growth factor therapy remains underexplored.

As a result, extrapolation from tendon-to-bone studies to muscle-to-bone healing is limited because interventions that improve tendon reattachment (e.g., growth factors or scaffolds) may not adequately support myofiber regeneration or functional integration at the bone interface. Muscle-to-bone repair typically requires approaches that support both myogenic regeneration and bone interface anchoring [296], whereas tendon-to-bone therapies mainly focus on collagenous tissue integration and enthesis formation [298].

### 7.3. BPC 157

The beneficial effect of BPC 157 as therapy after surgical detachment of the quadriceps muscle from its attachments, resulting in muscle-to-bone reattachment in rats [161], combines particular resolving of the most complex circumstances. Severe failure, new bone formation is absent, and an intervening connective tissue layer separates the muscle from the bone, including the whole system. Included are bones (ilium, femur, and tibia), tendons (all four parts of the quadriceps muscle ultimately inserted into the tuberosity of the tibia via the patella, where the quadriceps tendon becomes the patellar ligament), joints, neurovascular elements, and the muscles responsible for moving the legs [302] and the wide range of motions involved in standing and walking. Vice versa, with re-establishing all activities of the legs, the reattachment of the quadriceps muscles signifies that BPC 157 therapy successfully coordinated actions between the quadriceps muscles and other muscle groups. As a proof of concept of BPC 157 therapy, functional, ultrasonic, magnetic resonance, biomechanical, macroscopic, and microscopic effects were unmistakably attributed; muscle-to-bone reattachment occurred with effective resolution of a disruption of the periosteum, the vascular supply between the muscle and periosteum, and the interconnections between muscle and bone [161]. In addition, translational validity includes administration routes, which systematically evaluated the compound’s efficacy following oral route at multiple time points up to 90 days. This approach enhances the robustness of the data and contributes to a more comprehensive pharmacodynamic profile.

These points are summarized in Table 10.

## 8. Conclusions

*General conceptualization*. In conclusion, no previous review has systematically evaluated PRP, growth factors and BPC 157 efficacy for tendon–ligament–muscle and junction healing as cytoprotection therapies, preclinical to clinical evidence. Cytoprotection, healing to restore tissue integrity, outlines carrier-independent exogenous efficacy. The concept holds tendon, ligament, and muscle healing, in particular. Then, it holds their healing together, as interconnected lesions. Consequently, this review presents the possibilities for the cytoprotective therapies, suited for the tendon/ligament/muscle, and recovery of osteotendinous, myotendinous, and muscle-to-bone junction. The estimated key was the success of injury recovery amid each agent’s direct exogenous administration, alone or with a carrier, locally or systemically, without reliance on complex scaffolds, carriers, or tissue-engineering constructs. Notably, in comparison with growth factor therapy achievement [15,16,17,18,19,70,71,72,73,74,75,76,77,78,79], it can be suggested that achievement of BPC 157 therapy [13,14,15,16,17,18,19,20,21,22,23,43,44,54,55] is more pronounced. Likewise, BPC 157 therapy possesses full recovery, even if both remain mostly preclinical.

*Growth factors*, *PDGF*, *TGF-β1*, *IGF-1*, *FGF*, *VEGF*, *BMPs*. As reviewed, while with commonly acknowledged physiological significance, and acting throughout cytoprotection principles, growth factors (PDGF, TGF-β1, IGF-1, FGF, VEGF, BMPs), delivered locally with various carriers, improve tendon, ligament, and muscle healing; however, some (PDGF, TGF-β1, IGF-1) may fail in muscle lesions, and all show limited or no efficacy in junctional healing.

*Stable gastric pentadecapeptide BPC 157. General consideration.* Contrarily, proposed as a cytoprotection mediator, BPC 157 acts alone with a full cytoprotection range, given systemically or locally. Moreover, without any carrier, BPC 157 acts alone, combining beneficial effects on tendon, ligament, and muscle injuries with osteotendinous, myotendinous, and muscle-to-bone healing. In rat studies, across systemic (intraperitoneal, intragastric, or drinking water) and local (cream) administration, BPC 157 consistently demonstrated efficacy, indicating considerable translational potential.

*BPC 157. Specific consideration.* Therefore, the recovery of surgical detachment of the quadriceps muscle from its attachments, resolved reattachment by the BPC 157 therapy, finally concludes such coordinated action involving tendon [147,148,149,150,152,153], ligament [151], and muscle [200,211,212,213,214,215,216,217] injury, osteotendinous [147,148], myotendinous [160], and muscle-to-bone [161] junction therapy.

As emphasized above, considering its consistent high efficacy, whatever way of application, when administered as a native peptide (and thereby advantageous over growth factors that need carrier and local application), the stable gastric pentadecapeptide BPC 157 consistently promotes muscle healing and functional recovery [152,200,211,212,213,214,215], involving striated, smooth, and cardiac muscle [18,19]. It supports the simultaneous healing of different tissue types and facilitates the recovery of various junctional structures [147,148,160,161,223]. Along with muscle-to-bone healing [161], reported benefits include the restoration of neuromuscular [223], osteotendinous [147,148], and, particularly, myotendinous junctions [160]. Alongside enhanced muscle healing and functional recovery [147,148,149,150,151,152,153,160,161,200,211,212,213,214,215,216,217], BPC 157 also promotes the repair of adjacent structures that typically do not heal spontaneously. This includes the recovery of severely damaged tendons [147,148,149,153], ligaments [151], and bones [303,304,305,306]. Consequently, under BPC 157 treatment [13,14,15,16,17,18,19,20,21,22,23,43,44,54,55], bone and skeletal muscle can be reintegrated into functional units [307], as indicated with BPC 157 therapy, which effectively resolves complex muscle-to-bone reattachment in the most complex and demanding rat model study. Moreover, in accordance with its cytoprotection role, its strong wound-healing potential [13,43,44] supports recovery from diverse and otherwise refractory injuries, including crush [211,212], denervation [213,214,215], full transection [149,151,200], detachment [147,148,160,161], pseudoarthrosis [303], and bone loss [304,305]. In addition, there is recovery of various muscular weaknesses, peripheral [92,223,227,228,229,230,231,232,233,234,235,239,240,241,242] and central [214,215,236,237,238,245,246,247,248,249], including tumor-cachexia recovery [92]. Mechanistically, BPC 157 interacts with numerous molecular pathways [83,84,85,86,87,88,89,90,91,92], particularly those involving NO signaling [13,85,86,308], and functions as both a stabilizer of cellular junctions and a free radical scavenger [91,92].

Thus, we can conclude comparing growth factors (local, carriers)/BPC 157 (acting alone, systemically or locally) efficacy. It should be noted that, in rat studies, BPC 157 consistently demonstrated efficacy, indicating considerable translational potential. Further clinical studies will strengthen cytoprotective therapy and, particularly, BPC 157 in complex musculoskeletal and junctional injuries.

## 9. Limitations as Future Directions

*Growth factors and BPC 157 evidence.* Thus, the evidence was provided that highlights the therapeutic cytoprotection potential of BPC 157 in tendon, ligament, and muscle injuries as well as osteotendinous, myotendinous, and muscle-to-bone junction repair and compared with classical growth factors (PDGF, TGF-β1, IGF-1, FGF, VEGF, BMPs). More than growth factors (local application, carrier), these BPC 157 findings align with different delivery methods in one pathological model. BPC 157 studies have systematically evaluated the compound’s efficacy following systemic (oral, intraperitoneal, and intragastric) or local (cream) administration, often within the same pathological model. Therefore, similar effectiveness of different ways of administration can be a suggestive argument that translational validity stands. However, it may be that a rat model simplifies these variables, which is scientifically useful but limits direct clinical predictability.

*Breadth of BPC 157 efficacy.* Notably, the classical single-pathway pharmacological framework would recognize the reported breadth of BPC 157 efficacy as disproportionate. Contrarily, the cytoprotection-based perspective (i.e., preservation of cellular integrity, maintenance of microcirculatory function, redox homeostasis, and coordinated tissue repair across organ systems) means pleiotropic and multi-tissue effects. Therefore, the recovery of tendon, ligament, muscle, and their junctions is within this framework and reflects the integration of many processes (i.e., angiogenic, NO–system–modulating, junction-stabilizing, and anti-fibrotic processes) and not on a single dominant molecular target. Thus, the reproducibility of functional and structural recovery across diverse injury models supports biological plausibility rather than implausible over-efficacy. On the other hand, the precise molecular hierarchy governing these effects remains incompletely defined. In full implementation of the cytoprotection concept, as pointed out for BPC 157, a cytoprotection mediator, pleiotropic beneficial effects are anchored to its resolving effects on increased angiogenesis, increased VEGF, increased egr-1 gene, increased NO, or eNOS stimulation, and increased free radical formation. In this, the additional precise molecular targets and mechanisms of BPC 157 remain to be further defined. Notably, BPC 157 was pointed out as a therapy and safety key: a special beneficial pleiotropic effect controlling and modulating angiogenesis and the NO system [13] (i.e., BPC 157: corneal ulcer healing↑/corneal neovascularization↓ = tendon injury healing↑/promoted angiogenesis↑) [13]. In addition, BPC 157 therapy counteracts the adverse effects of corticosteroids [147,148,149,212] and NSAIDs [55]. On the other hand, taken together with the cytoprotection concept [1,2,3,4,5,6,7,8,9], as a concept still not implemented in clinics, taken as a hypothesis-based interpretative model (i.e., “ideal” agent), although elaborated with other agents found to share some of the cytoprotective properties, these could still oversimplify the multifaceted nature of musculoskeletal and junction disorders.

*The majority of experimental work.* The majority of experimental work (i.e., initial discovery, mechanistic exploration, and model validation) [13,14,15,16,17,18,19,20,21,22,23,43,44,54,55] is within a single research group. Importantly, this aligns with the confirmatory reports of other groups [82,83,84,85,86,87,88,89,90,308,309,310,311,312,313,314,315,316,317]. Additionally, this includes favorable safety reports [49,50,318] that are comparable with previous notations (for details, [13]). Illustratively, a novel study suggests that BPC 157 drives angiogenesis through FBXO22-dependent stabilization of BACH1 [319]. In a preprint report, Steven K. Schlosser suggested BPC 157 binding to SH# domains and the activation of Src family kinases.

*Perspective.* There is no substitute for rigorous clinical validation, which remains essential. Nevertheless, as discussed in our most recent review [320], cautiously drawn historical analogies—while inherently hypothetical—may be informative. Specifically, a concentration of early experimental work within a limited number of research groups has, in several instances, preceded successful clinical translation. Notably, similar developmental trajectories were observed during the early preclinical phases of several now-established therapies, including erythropoietin (EPO) [321,322], glucagon-like peptide-1 (GLP-1) analogs prior to industrial expansion [323,324,325], and neuropeptides such as pituitary adenylate cyclase-activating polypeptide (PACAP) [326] and vasoactive intestinal peptide (VIP) [327,328].

*Clinical data.* In humans, regardless of substantial global attention and off-label use, given still limited clinical data [45,46,47,48,50], long-term safety, optimal dosing, and pharmacokinetic profiles are not yet fully characterized. Nevertheless, oral bioavailability, prophylactic and therapeutic efficacy, and safety in preclinical models and in limited trials make it attractive for further translational research. Future studies should prioritize well-controlled clinical trials and mechanistic investigations to clarify its role as a cytoprotective therapy for musculoskeletal and junctional injuries.

Consequently, the native peptide therapy, with cytoprotective pentadecapeptide BPC 157 (systemic (including per-oral) and local (cream) application), which is known to be stable in human gastric juice, could provide a beneficial effect in tendon, ligament, muscle, and bone healing [13,14,15,16,17,18,19,20,21,22,23,43,44,54,55,219]; this can be effectively combined to re-establish tendon–bone, tendon–muscle, and muscle–bone reattachment. In this way, its effects, i.e., functional, ultrasonic, magnetic resonance, biomechanical, macroscopic, and microscopic effects, were unmistakably attributed, which would clearly demonstrate defined healing in further practice.

## Figures and Tables

**Table 1:** Comparative delivery, functional healing evidence, side effects, and translational status for growth factors, PDGF, TGF-beta, IGF-1, bFGF, BMPs, and BPC 157 in tendon healing.

Features	Classic Growth Factors PDGF, TGF-Beta, IGF-1, bFGF	BMPs (GDP 5–14)	BPC 157
Delivery	Local, short half-life; requires carrier (fibrin/gel)	Local scaffold or gene vector; risk of ossification	Systemic or local in saline; stable without carrier
Functional healing evidence	Mostly surrogate histology, limited biomechanical proof	Preclinical only; risk of ectopic ossicle	Full biomechanical and functional recovery in rats (Achilles, rotator cuff, tendon-to-bone)
Side effects	Excess angiogenesis, fibrosis, and short-lived effect	Ossicle formation, heterotopic bone	No ossicle formation, balanced angiogenesis, cytoprotection
Translational status	Investigational; variable efficacy in humans	Preclinical; safety concern	Robust preclinical efficacy; human trials lacking

**Table 2:** Tendon injury recovery with exogenous administration of growth factors and the supposed main mechanism, via delivery system and carrier, to the key outcome, based on the presented references.

Growth Factor	Main Mechanism	Delivery/Carrier	Key Outcome	References
**PDGF-BB**	Stimulates fibroblast proliferation, collagen remodeling	Novel fibrin/heparin-based delivery system for sustained release at repair site	Enhanced fibroblast proliferation and collagen remodeling in canine flexor tendon repair	Thomopoulos et al., 2015 [110]
**PDGF-BB**	Enhances tendon cell proliferation in vitro	Direct culture of tendon cells	Increased proliferation of tendon fibroblasts	Liang et al., 2009 [111]
**TGF-β1**	Promotes collagen synthesis and cell proliferation	Adenovirus-modified muscle grafts, localized delivery	Improved tendon healing in rat model	Majewski et al., 2012 [112]
**TGF-β3**	Enhances collagen organization, regulates adhesion formation	Nanoparticles for local release in the rat Achilles Tendon	Improved tendon healing with reduced adhesion formation	Cetik et al., 2022 [113]
**TGF-β3**	Regulates adhesion formation via JNK/c-Jun pathway	Adenoviral-mediated overexpression (ad-TGF-β3) in rat flexor tendon	Reduced adhesions, modulated tendon repair	Jiang et al., 2021 [114]
**TGF-β (general)**	Dual role: promotes repair (collagen synthesis, proliferation) but may induce fibrosis/adhesions if uncontrolled	Review	Highlights the importance of controlled delivery	Li et al., 2022 [115]
**IGF-I**	Stimulates collagen synthesis, fibroblast proliferation, angiogenesis	Collagen sponge applied locally to tendon injury site	Accelerated functional recovery in rat Achilles tendon repair	Kurtz et al., 1999 [116]
**IGF-I**	Enhances cellular/molecular aspects of tendon healing	Intratendinous injection in collagenase-induced flexor tendinitis (horse)	Improved cellular proliferation, collagen synthesis	Dahlgren et al., 2002 [117]
**IGF-I**	Stimulates tendon collagen synthesis in humans	Local administration	Increased collagen synthesis in patellar and Achilles tendons	Hansen et al., 2013 [118]
**IGF-I**	Investigated in clinical trial for chronic tendinopathy	Intratendinous injection + heavy slow resistance training	No superior structural or clinical outcomes compared to control	Olesen et al., 2021 [119]
**bFGF/FGF-2**	Stimulates type III collagen synthesis, cell proliferation	Bolus intratendinous injection [120,121]; FGF-2 coated nanofiber scaffold [122]	Early increase in cell proliferation and collagen III; scaffold delivery improved tissue organization and fibril alignment	Chan et al., 2000 [120] Kraus et al., 2016 [121] Turgut et al., [122]

**Table 3:** Summary table of BMPs studied in tendon healing, incorporating isoform, tendon effect, delivery method, and risk of ossicle formation, based on preclinical studies.

BMP Isoform	Tendon Effect	Delivery Method/Carrier	Risk of Ossicle Formation	References
**BMP-2**	Promotes collagen synthesis, fibroblast proliferation, tendon matrix remodeling	Local injection, collagen sponge, or fibrin gel	High; heterotopic ossification and ectopic ossicle formation reported	Wang et al., 2012 [140]
**BMP-7** **(OP-1)**	Stimulates tenogenic differentiation, collagen production	Local implantation via scaffold or carrier	Moderate; ossicle formation possible at high doses	Friedlaender et al., 2001 [141]
**BMP-12 (GDF-7)**	Enhances tenogenic differentiation, fibroblast proliferation, tendon matrix organization	Local application via collagen-coated sutures or adenoviral gene transfer	Low; generally tenogenic with minimal ossification	Wolfman et al., 1997 [142]
**BMP-13 (GDF-6)**	Supports early tendon healing and matrix deposition, promotes tenogenic differentiation	Local injection or gene transfer in animal models	Low; minimal ossicle formation reported	Wolfman et al., 1997 [142]
**BMP-14 (GDF-5)**	Promotes collagen synthesis, fibroblast proliferation, tendon remodeling	Local injection or scaffold-based delivery	Low to moderate; dose-dependent risk	Lou et al., 2001 [143]

**Table 4:** Table summarizing the BPC 157 tendon studies that specifically address tendon injury/healing (in vitro, ex vivo, and in vivo), all the key details: model, route/dose, and main tendon-specific outcomes.

Citation	Model/Injury	Route, Dose (as Reported)/Carrier	Main Outcomes (Tendon Specific)
Staresinic et al., 2003 [149]	Rat—full transection of Achilles tendon; in vitro tenocyte	Intraperitoneal (i.p.) daily; BPC 157 in saline, doses: 10 µg/kg, 10 ng/kg, 10 pg/kg	Accelerated functional recovery (higher Achilles functional index (AFI)), improved biomechanical properties (load to failure, Young’s modulus), superior histology (organized fibroblasts, collagen), closed defect; in vitro tenocyte survival/growth enhanced
Krivic et al., 2006 [147]	Rat—Achilles tendon sharply detached from calcaneus (tendon → bone defect)	i.p. daily; BPC 157 in saline; doses: 10 µg/kg, 10 ng/kg, 10 pg/kg; with/without methylprednisolone	Improved functional recovery (AFI), enhanced biomechanical metrics (load to failure, stiffness, Young’s modulus), better collagen organization, BPC 157 opposed steroid-induced impairment
Krivic et al., 2008 [148]	Rat—Achilles tendon → bone transection (early recovery phase days 1–4)	i.p. daily; BPC 157 10 µg/kg; compared to methylprednisolone 5 mg/kg and saline	Increased early AFI (improved early functional recovery), decreased MPO activity, reduced inflammatory cell influx, increased early neovascular index; methylprednisolone impaired angiogenesis and functional recovery
Chang et al., 2011 [83]	Rat—ex vivo tendon explants; primary cultured tendon fibroblasts (Achilles)	In vitro/ex vivo; BPC 157 applied at multiple concentrations	Accelerated tendon explant outgrowth, increased fibroblast migration and spreading, enhanced cell survival under oxidative stress, induced F-actin formation, activated FAK–paxillin signaling
Chang et al., 2014 [84]	Rat tendon fibroblasts in vitro	In vitro; BPC 157 at concentrations tested in culture	Increased expression of growth hormone receptor in tendon fibroblasts, suggesting a mechanism for tendon anabolic effects
Sikiric et al., 2014 [153]	Rat—surgical detachment of supraspinatus and infraspinatus (rotator cuff tear)	i.p. daily; BPC 157 10 µg/kg	Near-complete functional recovery, tendon healing of supraspinatus/infraspinatus, restored mobility, muscle strength, leg length

**Table 5:** Comparison of classical growth factors and BPC 157 in tendon healing.

Agent	Primary Mechanism(s)	Target/ Effects	Delivery Method	Limitations/ Risks	Distinctive or Potential Advantages of BPC 157
PDGF-BB	Chemotaxis, fibroblast proliferation, matrix synthesis	Recruitment of tendon stem/ progenitor cells; collagen deposition	Local (fibrin gel, injection)	Limited mechanical gain; transient effect	BPC 157 induces sustained fibroblast activation and collagen maturation without carrier dependence
TGF-β1	ECM synthesis, scar formation, myofibroblast differentiation	Early granulation, collagen I/III ratio modulation	Local injection/ scaffold	Risk of fibrosis, adhesions	BPC 157 promotes orderly collagen repair while limiting scar hypertrophy
IGF-1	Anabolic, stimulates collagen synthesis and tenocyte proliferation	Enhances fibroblast activity and tensile strength	Local injection	Short half-life; variable human efficacy	BPC 157 shows prolonged bioactivity and systemic reparative capacity
bFGF (FGF-2)	Angiogenesis, fibroblast proliferation	Accelerates early healing	Local injection	Excess angiogenesis; inferior long-term alignment	BPC 157 provides balanced angiogenesis and superior tensile remodeling
VEGF-A/VEGF-111	Vascularization, endothelial proliferation	Promotes neovessel formation and nutrient delivery	Local (gel, injection)	Over-vascularization → impaired collagen organization	BPC 157 enhances microcirculation while preventing excessive neovascularization
BPC 157	NO modulation, cytoprotection, balanced angiogenesis, fibroblast proliferation, anti-inflammatory action	Enhanced collagen organization, angiogenesis normalization, tendon–muscle continuity	Systemic or local; no carrier required	Clinical validation ongoing	Pleiotropic; coordinates angiogenesis, inflammation, and matrix remodeling—comprehensive tissue restoration

**Table 6:** Growth factors in ligament healing (medial collateral ligament (MCL), anterior cruciate ligament (ACL)), efficacy, carriers, and outcomes.

Study	Growth Factor(s)	Dose/ Concentration	Carrier	Model (In Vitro/In Vivo)	Key Outcomes
Schmidt et al., 1995 [170]	EGF, bFGF, PDGF-BB	0.1–10 ng/mL	None	In vitro (rabbit MCL and ACL fibroblasts)	Dose-dependent fibroblast proliferation; bFGF and PDGF-BB most potent; ACL fibroblasts less responsive than MCL.
Lee et al., 1995 [171]	EGF, PDGF, bFGF, TGF-β	Varied	None	In vitro (rabbit MCL and ACL explants)	Synergistic effect on fibroblast outgrowth with combined growth factors.
Woo et al., 1998 [172]	PDGF-BB, TGF-β, EGF	Various doses	Fibrin sealant	In vivo (rabbit MCL)	Dose-dependent biomechanical improvement in MCL healing; enhanced tensile strength and collagen organization.
Hildebrand et al., 1998 [173]	PDGF-BB	10 µg	Fibrin sealant	In vivo (rabbit MCL)	Increased ultimate load and energy absorption; improved histologic healing quality.
Scherping et al., 1997 [174]	IGF-1 bFGF, PDGF-AA	1.0 ng/mL	None	In vitro (rabbit MCL fibroblasts)	Significant stimulation of fibroblast proliferation and matrix synthesis.
Spindler et al., 2003 [175]	TGF-β1	7 µg	Carrier solution	In vivo (rabbit MCL)	No early biomechanical improvement; monotherapy insufficient for functional healing.
Sakai et al., 2002 [176]	EDF TGF-β1	Various doses	Fibrin sealant	In vivo (rabbit MCL)	Improved histological and mechanical parameters in a dose-dependent manner.
Marui et al., 1997 [177]	Basic FGF, acidic FGF, TGF-β1, and EGF	Varied	None	In vitro (ligament fibroblasts)	Enhanced collagen and proteoglycan synthesis; supports matrix regeneration.
Nagumo et al., 2005 [178]	TGF-β1, EGF PDGF-BB	4 ng TGF-β1, 20 ng EGF, and 4 µg PDGF-BB	Fibrin sealant	In vivo (rabbit ACL)	The TGF-β1 group showed significantly better tensile strength and tangent modulus compared to sham, though still below the normal ACL. The EGF and PDGF-BB groups did *not* show significant improvement.
Hee et al., 2012 [179]	PDGF	—	—	Review/ Preclinical PDGF	The outcomes of the preclinical studies reviewed here strongly suggest that rhPDGF-BB will provide a new therapeutic opportunity to improve the treatment of injured tendons and ligaments.

**Table 7:** Classical growth factors vs. BPC 157 in muscle injury therapy (with carriers and application routes).

Factor	Mechanism	Carrier/ Formulation	Application Route	Key Outcomes	References
**IGF-1**	Promotes myoblast proliferation, muscle hypertrophy, and Angiogenesis	Recombinant protein	Intramuscular injection	Improved muscle size and function in ischemic limbs	Dong et al., 2023 [189]
**IGF-1**	Activates satellite cells, promotes myogenesis	Engineered mRNA	Local injection	Enhanced regenerative potential in vivo, improved fiber formation	Antony et al., 2023 [190]
**FGF-2**	Stimulates satellite cell proliferation and differentiation	Recombinant protein	Intramuscular injection	Accelerated muscle regeneration	Lefaucheur and Sebille, 1995 [191]
**FGF**	Satellite cell recruitment, proliferation	Recombinant protein	Intramuscular injection	Enhanced satellite cell recruitment in young and old rats	Yablonka-Reuveni et al., 1999 [192]
**FGF-2**	Enhances myoblast proliferation, supports functional recovery	Recombinant protein	Intramuscular injection	Improved functional recovery of reinnervated muscle	Iwata et al., 2006 [193]
**FGF-2**	Overexpressed in myoblasts; reduces apoptosis, increases proliferation	Alginate-encapsulated myoblasts	Transplantation into injured muscle	Enhanced regeneration, reduced apoptosis	Stratos et al., 2011 [194]
**HGF**	Activates satellite cells, modulates macrophage phenotype	Recombinant protein/plasmid	Intramuscular injection	Promoted M1→M2 macrophage transition, enhanced regeneration	Choi et al., 2019 [195]
**HGF**	Satellite cell activation and differentiation	Recombinant protein	Intramuscular injection	Affected satellite cell activation, mixed effects on regeneration	Miller et al., 2000 [196]
**VEGF-A**	Promotes angiogenesis, improves vascularization	AAV vector	Intramuscular injection	Enhanced muscle fiber regeneration and vascularization	Arsic et al., 2004 [197]
**VEGF**	Angiogenesis, reduces fibrosis	Collagen matrix coated with VEGF	Local application	Improved muscle force recovery, reduced scar formation	Frey et al., 2012 [198]
**VEGF**	Vascularization, stem cell support	VEGF-expressing MDSCs	Transplantation	Enhanced vascularization, muscle fiber regeneration in dystrophic muscle	Deasy et al., 2009 [199]
**BPC** **157**	Cytoprotective, promotes angiogenesis, modulates growth factor expression	—	Intraperitoneal injection	Accelerated muscle fiber healing, reduced inflammation, improved functional recovery	Staresinic et al., 2006 [200]

**Table 8:** BPC 157 muscle therapy.

Model/ Injury Type	Key Study	Administration and Dose	Main Histologic Outcomes	Main Functional/ Biomechanical Outcomes	Mechanistic Insights/Notes
Full quadriceps transection	Staresinic et al., 2006 [200]; Brcic et al., 2009 [152]	Intraperitoneal 10 μg, 10 ng, 10 pg/kg/day	Accelerated myofiber regeneration, reduced necrosis and fibrosis, enhanced early angiogenesis, regained muscle continuity	Faster recovery of limb function and muscle strength compared with untreated controls	Promotes early vascularization and matrix deposition; supports satellite cell activation
Muscle crush Injury	Novinscak et al., 2008 [211]	10 μg, 10 ng/kg/day intraperitoneally, BPC 157 locally as 1.0 μg or 0.01 μg dissolved in distilled water per gram of commercial neutral cream/day	Reduced tissue necrosis, increased angiogenesis, improved myofiber organization	Improved functional recovery and strength compared with untreated controls	BPC 157 protects against ischemic and mechanical tissue stress; modulates inflammatory response
Corticosteroid-impaired muscle healing	Pevec et al., 2010 [212]	10 μg, 10 ng/kg/day intraperitoneally, BPC 157 locally as 1.0 μg or 0.01 μg dissolved in distilled water per gram of commercial neutral cream/day	Counteracted steroid-induced myofiber degeneration; increased vascularization	Restoration of functional recovery partially blocked by corticosteroids is rescued	Cytoprotective and angiogenic effects overcome steroid-induced healing inhibition
Denervated Muscle	Mihovil et al., 2009, [213]	Intraperitoneal 10 μg/kg/day	Reduced atrophy, enhanced myofiber regeneration, increased capillary density	Improved muscle contractility and partial restoration of function	May support neurotrophic and angiogenic pathways; promotes tissue survival in absence of normal innervation
Hindlimb ischemia/ vascular impairment	Hsieh et al., 2017 [86]	Intraperitoneal 10 μg/kg/day	Enhanced angiogenesis, VEGFR2 upregulation, improved capillary perfusion	Improved limb perfusion and muscle endurance	VEGFR2-dependent pro-angiogenic signaling; improved microcirculation
Spinal cord Injury (SCI)/ secondary muscle injury	Perovic et al., 2019 [214]; Perovic et al., 2022 [215]	Intraperitoneal 200 or 2 μg/kg; Intraperitoneal 2 μg/kg, Peroral 10 μg/kg/day	Reduced muscle atrophy secondary to SCI, increased microvascular density, preserved myofiber morphology	Improved locomotor function, gait, and hindlimb coordination, recovered tail paralysis	Supports angiogenesis and neuroprotective effects; reduces secondary degeneration; facilitates functional recovery

**Table 9:** Osteotendinous junction therapy.

Study	Model (Species/Injury)	Agent Tested	Application Method and Carrier	Key Outcomes
Rodeo et al., 1993 [270]	Dog—tendon graft in drilled bone tunnel (classic tendon → bone model)	—(baseline/ model paper)	Surgical tendon transplantation into bone tunnel; histologic/biomechanical follow-up (no exogenous GF/BMP)	Described timetable and histologic sequence of tendon-to-bone healing (fibrovascular interface → Sharpey-like fibers → gradual osseous incorporation). Widely used reference model for augmentation studies.
Seeherman et al., 2008 [272]	Sheep—acute rotator-cuff repair model	Recombinant human BMP-12 (rhBMP-12/GDF-7)	Local delivery at repair site; compared different carriers (collagen sponge vs. hyaluronan paste in preclinical work); rhBMP-12 delivered on carrier placed at tendon → bone repair	Accelerated early healing, increased load-to-failure and improved early enthesis formation versus control (carrier mattered for retention).
Lee-Barthel et al., 2018 [273]	Engineered bone-to-bone ligament enthesis (ACL/enthesis model)—ex vivo/in vivo testing	BMP-4 (localized release)	Localized BMP-4 release from brushite cement anchors (designed for local, sustained release at the enthesis)	Improved enthesis formation: increased enthesis-related gene expression (Sox9, aggrecan, tenascin C, osteopontin) and higher interface mechanical strength (improved failure load).
Kabuto et al., 2015 [274]	Rat—rotator cuff tendon → bone repair	BMP-7	BMP-7 loaded onto a gelatin hydrogel sheet applied to tendon insertion for sustained local release	Sustained BMP-7 release from GHS improved tendon-to-bone healing (histology) compared with bolus and increased markers of fibrocartilage/repair.
Ozeki et al., 2013 [275]	Rat—Achilles tendon models (tendon manipulation/ transplantation)	BMP-7	Local injection of BMP-7 into tendon tissue (in situ injections) prior to transplant/implantation	BMP-7 modulated matrix gene expression and promoted tissue changes consistent with enhanced matrix production/repair in tendon tissue used for reconstruction/ transplantation contexts.
Krivic et al., 2006 [147]	Rat—Achilles tendon detachment from calcaneus (tendon-to-bone detachment model)	BPC 157	Systemic administration (reported intraperitoneal dosing in the paper) given after detachment; no additional carrier required	BPC 157 promoted functional recovery (Achilles functional index), improved biomechanical properties (higher load-to-failure, stiffness), better collagen organization and vascularization; it opposed corticosteroid-induced aggravation of tendon-to-bone healing.
Krivic et al., 2008 [148]	Rat—Achilles tendon → bone transection model	BPC 157 ± methylprednisolone (comparator)	Systemic (reported intraperitoneal) administration; compared BPC 157, methylprednisolone, combination	BPC 157 improved early functional recovery versus steroid; it countered steroid-induced delay in functional/biomechanical healing parameters.
Sikiric et al., 2014 [153]	Rat—rotator cuff tear model	BPC 157	Reported systemic administration—peptide given after experimental rotator cuff tear	Abstract reports improved healing and functional recovery in BPC 157 treated rats—supports preclinical efficacy in rotator cuff model.
Rodeo et al., 1999 [154]	Review—rotator cuff repair models (preclinical + clinical)	Growth factors/BMPs/cells/ carriers (overview)	Review summarizing delivery strategies (collagen sponge, hydrogels, anchors, local injections) and outcomes	Review summarizing delivery strategies (collagen sponge, hydrogels, anchors, local injections) and outcomes.

**Table 10:** Summarized comparison: BPC 157 therapy and growth factor therapy achievement (N/A—not applied).

Injury/ Junction	Classical Growth Factors	Administration /Carrier	Key Outcomes	Limitations	BPC 157	Administration	Key Outcomes	Advantage
**Ligament injuries**	**PDGF,** **IGF-1,** **FGF,** **VEGF**	Local, carrier-dependent (hydrogels, sponges)	Partial collagen deposition, angiogenesis	Local effect only, junction recovery limited	**BPC 157**	Systemic (IP, oral) or local cream	Full ligament fiber restoration, angiogenesis, functional recovery	Carrier-free, systemic, consistent, functional recovery
**Tendon injuries**	**PDGF,** **IGF-1,** **FGF,** **VEGF**	Local delivery with carriers	Enhanced early healing, some collagen alignment	Limited functional recovery, local only	**BPC 157**	Systemic (IP, oral) or local cream	Improved collagen orientation, vascularization, functional recovery	Systemic efficacy, pleiotropic cytoprotection, analgesic effect
**Muscle** **injuries**	**IGF-1, FGF-2, HGF, VEGF,** **TGF-β1**	Local (IM, viral vectors, gels, hydrogels)	Myofiber proliferation, angiogenesis	Carrier needed, limited systemic effect, scar tissue formation	**BPC 157**	Systemic (IP, oral) or local cream	Myofiber regeneration, angiogenesis, reduced fibrosis, restored function	Systemic, carrier-free, neuromuscular protection, steroid/NSAID counteraction
**Osteotendinous junction (OTJ)**	**BMP-2/4/7/12, FGF-2**	Local, sustained release (hydrogel, sponge, cement)	Fibrocartilage formation, load-to-failure improvement	Carrier dependent, no systemic therapy, functional recovery limited	**BPC 157**	Systemic (IP)	Improved collagen alignment, vascularization, biomechanics, counteracts corticosteroid impairment	Carrier-free, systemic, functional recovery, supports tendon–muscle–bone integration
**Myotendinous junction (MTJ)**	**IGF-1,** **FGF,** **TGF-β, BMPs**	Mechanistic, exercise or developmental; no direct exogenous therapy	N/A (no verified repair studies)	No direct therapy studies	**BPC 157**	Systemic (IP, oral)	Full restoration of MTJ, muscle–tendon continuity, functional and biomechanical recovery, anti-inflammatory and angiogenic effects	First and only verified therapy for MTJ, systemic and carrier-free
**Muscle-to-bone**	**None** **directly studied**	N/A	N/A	No preclinical studies; tendon-to-bone data not fully translatable	**BPC 157**	Systemic (oral, IP)	True reattachment of muscle to bone, histologic fiber penetration, periosteal reactivation, bone remodeling, leg function normalized	First verified therapy for complex muscle-to-bone reattachment, systemic, functional recovery, multi-junction integration

## Data Availability

No new data were created or analyzed in this study. Data sharing is not applicable to this article.
